# Cytogenetic analysis in *Thoracocharax stellatus* (Kner, 1858) (Characiformes, Gasteropelecidae) from Paraguay River Basin, Mato Grosso, Brazil

**DOI:** 10.3897/CompCytogen.v6i3.3637

**Published:** 2012-09-26

**Authors:** Edson Lourenço da Silva, Rafael Splendore de Borba, Liano Centofante, Carlos Suetoshi Miyazawa, Patrícia Pasquali Parise-Maltempi

**Affiliations:** 1 Instituto de Biociências, UNESP Univ Estadual Paulista, Campus de Rio Claro, Departamento de Biologia, Laboratório de Citogenética. Av. 24A, 1515. CEP: 13506-900. Rio Claro, SP, Brazil; 2 Instituto de Biociências, UFMT Univ Federal de Mato Grosso, Departamento de Biologia e Zoologia. Laboratório de Citogenética Animal. Av. Fernando Corrêa da Costa s/n, CCBS-II, CEP: 78060-900. Cuiabá, MT, Brazil; 3 Universidade Federal do ABC, Centro de Ciências Naturais e Humanas (CCNH). Rua Santa Adélia, 166. Bairro Bangu, CEP 09.210-170. Santo André, SP, Brazil

**Keywords:** *Thoracocharax stellatus*, Ag-NOR, C-band, FISH, 18S rDNA

## Abstract

*Thoracocharax stellatus* (Characiformes, Gasteropelecidae) is a small Neotropical species of fish, widely distributed in several rivers of South America. Evidence for karyotype heteromorphysm in populations from different geographical regions has been reported for this species. In this way, populations of *Thoracocharax stellatus* from the Paraguay River basin were cytogenetically characterized and the results were compared with other studies performed in the same species but from different basins. The results showed a diploid number of 2n = 54 for *Thoracocharax stellatus*, with chromosomes arranged in 6 metacentric (m), 6 submetacentric (sm), 2 subtelocentric (st) and 40 acrocentric (a), for both sexes, with a simple Nucleolus Organiser Region (NOR) system reported by the techniques of silver nitrate impregnation and fluorescent *in situ* hybridisation (FISH) using 18S rDNA sequences as probe. The distribution of constitutive heterochromatin, observed by the C-band technique and Chromomycin A3 staining showed great similarity among the analyzed populations and consists mainly of discrete blocks in the pericentromeric and telomeric regions of most chromosomes. The presence of female heterogamety was also observed indicating a ZZ/ZW system with W chromosome almost totally heterochromatic. The results also show cytogenetic diversity of the group and are useful to understand the mechanisms of karyotype evolution of the family.

## Introduction

The family Gasteropelecidae (Characiformes) comprises a group of small Neotropical fishes that inhabit rivers of the main Central and South America basins, with exception of the southeast of Brazil and Chile (Géry 1977, Weitzman and Palmer 2003). Three genera are recognized in this family (*Carnegiella* Eigenmann, 1909, *Gasteropelecus* Scopoli, 1777 and *Thoracocharax* Fowler, 1906), comprising a total of nine nominal species.

The first taxonomic study regarding this family was conducted by Weitzman (1954, 1960) which suggested, by osteological observation, the genus *Thoracocharax* represents an independent lineage inside the group, named as tribe Thoracocharacini. The genera *Gasteropelecus* and *Carnegiella* comprise a sister group belonging to a second lineage named tribe Gasteropelecini (Weitzman 1960).

Despite the reduced number of species, this family presents many taxonomic problems related to the difficulty of finding species-specific characters. *Carnegiella marthae* (Myers, 1927) e.g. have osteological differences related to anal fin rays among specimens from Orinoco and Negro rivers, and the specimens from Peruvian Amazon and Madeira Rivers (Géry 1977). So the typical form from Orinoco-Negro has been called *Carnegiella marthae marthae*, and the species from Peruvian Amazon called *Carnegiella marthae schereri* (Géry 1977).

Thus, considering its large distribution, an accurate analysis of specimens from different localities can reveal the existence of putative new species (Weitzman and Weitzman 1982, Weitzman and Palmer 1996, Weitzman and Palmer 2003).

As regards to the cytogenetic aspects, the family Gasteropelecidae is relatively non-conserved, with diploid numbers varying from 2n=48 to 2n=54. The species studied until now are recognized by the presence of many subtelocentric and acrocentric chromosomes and almost all presenting one chromosome pair carrier the NOR, that can be variable only in some populations (Carvalho et al. 2002). Also been described and characterized in the family the sexual system ZZ/ZW for the genera *Carnegiella* and *Thoracocharax*.

The genus *Thoracocharax* is the most widely distributed among the gasteropelecids, and is characterized by species that have distinct chromosomal and morphological characteristics in different geographic regions. *Thoracocharax stellatus* (Kner, 1858),for example, presents a considerable morphological polymorphism among populations, mainly due to the geographic isolation (Silva et al. 2009)

The karyotypic diversity observed in *Thoracocharax stellatus* comprises different diploid numbers and polymorphism in NOR (Nucleolus Organiser Region) number and different chromosomes involved in the sexual differentiation (ZZ/ZW system) (Carvalho et al. 2002, Venere et al. 2007). These variations can help determining the taxonomic state of some populations.

Due to the cytogenetic diversity already observed for different *Thoracocharax stellatus* populations, the objective of this study was to describe the karyotypic structure of three populations from the Paraguay basin not studied yet and identify chromosomal markers for this species by establishing chromosomal variation patterns among different populations.

## Material and methods

**Figure 1.**
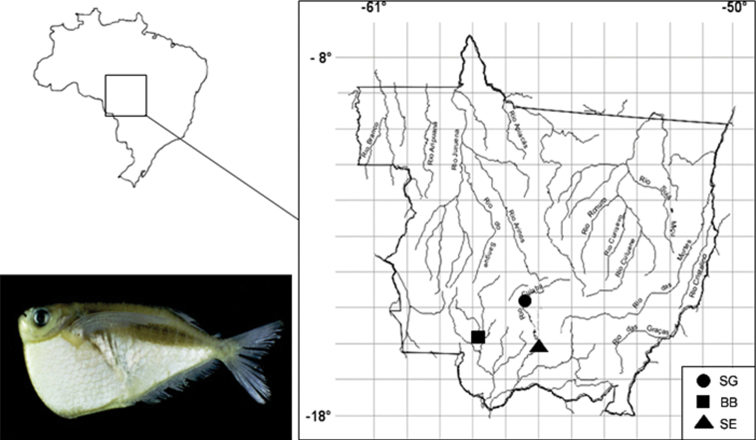
Sampling sites of the three populations of *Thoracocharax stellatus*. SG: São Gonçalo; SE: SESC and BB Barra do Bugres.

For the cytogenetic analysis of *Thoracocharax stellatus*, 111 specimens collected in the Paraguay basin were used, 38 from Cuiabá River - São Gonçalo (SG) (15°39'9.96"S, 56°4'8.62"W), 26 in Cuiabá River - SESC (SE) (16°38'55.0"S, 56°28'06.2"W) and 47 from Barra do Bugres (BB) (15°4'41.13"S, 57°10'55.64"W) (Fig. 1). The material collected was deposited in the Laboratório de Citogenética Animal/UFMT, Mato Grosso (LCA 018, LCA 023, LCA 028).

Direct cytological preparations were produced from kidney samples according to the methods given by Foresti et al. (1981). Karyotype analysis was conducted after conventional staining with Giemsa.

The chromosomes were morphologically classified according to the nomenclature proposed by Levan et al. (1964), with the following modifications: metacentric (m), submetacentric (sm), subtelocentric (st), and acrocentric (a). Heterochromatic regions were visualized by C-banding (Sumner 1972) and double fluorochrome staining (Chromomycin A3 + 4,6 – diamide 2 phenylindole - DAPI), according to the methods used by Schweizer (1980) and NORs were detected by silver nitrate impregnation (Ag-NORs), according to the procedure described by Howell and Black (1980).

The Fluorescent *in situ* hibridization (FISH) analyses were performed basically according to the method of Pinkel et al. (1986) with some modifications according to Silva et al. (2012). The 18S rDNA probe was obtained by PCR using specific primers set (NS1 5’-GTAGTCATATGCTTGTCTC-3’ and NS2 5’-GGCTGCTGGCACCAGACTTGC-3’) and labelled with digoxigenin by PCR. Metaphase chromosome slides were incubated with RNAse (40 μg/ml) for 1 h at 37°C and dehydrated using an ethanol series (70%, 85%, 100%). Chromosomal DNA was denatured for 1 min and 45 seconds in 70% formamide/2× SSC at 70°C and the spreads were dehydrated using the same ethanol series. The hybridisation solution (50% formamide/2× SSC, 10% dextran sulfate, and 1.5 μg/ml DNA probe) was denatured for 10 min at 95°C and applied to each slide under a coverslip. The hybridisation was performed overnight at 37°C in a moist chamber containing 2× SSC. Post-hybridisation washes were conducted using: 50% formamide/2× SSC (pH 7.0) twice for 5 min at 45°C; 2× SSC at 45°C; and 2× SSC at room temperature. Signals were detected using antidigoxigenin-rhodamine antibody. Chromosomes were counterstained with DAPI (1.5 μg/mL) and mounted in antifading solution.

Chromosomes were observed using an Olympus BX51 microscope coupled to an Olympus digital camera model D71. Chromosome images were captured using the DP Controller software.

## Results

**Figure 2.**
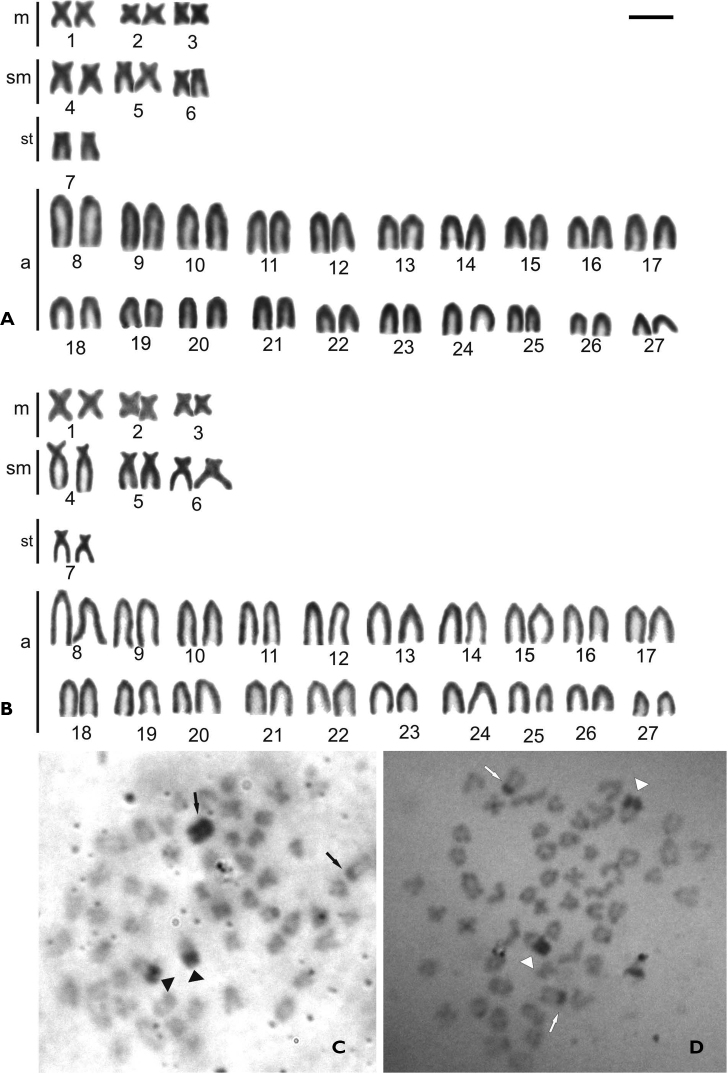
Female (**A**) and male (**B**) karyotype of *Thoracocharax stellatus* stained with Giemsa showing 2n=54 chromosomes. Metaphases from female (**C**) and male (**D**) of *Thoracocharax stellatus* showing heterochromatic blocks after C-band treatment. Arrows indicate the sex chromosomes Z and W in females and males Z and Z; arrowheads indicate the NOR chromosome. Bar = 10 µm.

Table 1 summarizes the karyotypic data obtained in the present study as well as in the available literature. The *Thoracocharax stellatus* specimens from three localities of the Paraguay River basin present diploid number 2n=54 chromosomes, with fundamental number NF=68 and karyotypic formulae that includes 6m, 6sm, 2st and 40a for both sexes (Figure 2 A, B).

**Table 1.** Chromosomal data on Gasteropelecidae from different Brazilian hydrographic basins.

					**karyotypic formula**				
**Species**	**Hydrographical basin**	**Sex chromosomes**	**Ag-NOR**	**2n**	**m**	**sm**	**st**	**a**	**Ref**
*Thoracocharax stellatus*	Araguaia	ZZ/ZW	2–4	54	6	6	6	36	1
*Thoracocharax stellatus*	Paraguay	ZZ/ZW	2	54	6	6	2	40	2
*Thoracocharax* cf. *stellatus*	Amazon	ZZ/ZW	2 ▲	52	8	16	4	24	3
*Carnegiella marthae*	Amazon	ZZ/ZW	2	50	20	12	4	14	4
*Carnegiella strigata*	Amazon	-	2–4	50	4	4	2	40	4

▲= Presence of size heteromorphisms. References= 1-Venere et al. (2007), 2- present paper, 3- Carvalho et al. (2002), 4- Terêncio et al. (2008)

The distribution pattern of constitutive heterochromatin presents similarity for all populations and is composed by discrete blocks mainly at the telomeric and pericentromeric regions in the majority of the chromosomes (Figure 2 C, D). The only exception was one acrocentric chromosome totally heterochromatic present in female karyotype, while the other chromosome of the pair presents a remarkable marker only in the pericentromeric region.

The male individuals present the conspicuous heterochromatin blocks in the pericentromeric region in corresponding chromosomes, indicating the presence of ZZ/ZW chromosome sex system in the studied populations (Figure 2 C, D).

**Figure 3.**
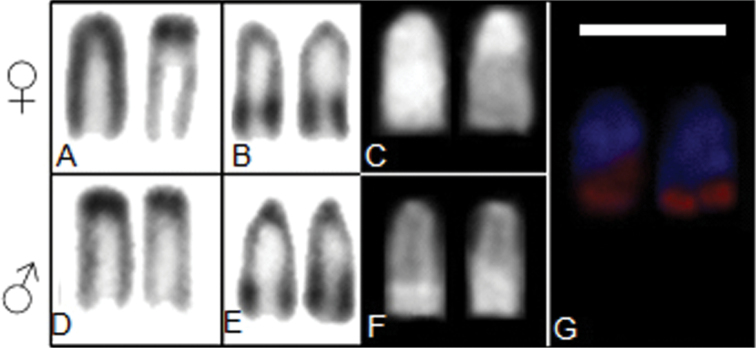
Chromosome markers from males and females individuals of *Thoracocharax stellatus* **A** C-band of Z and W chromosomes of female **B** Ag-NOR bearing chromosomes of female individuals **C** CMA_3_ treatment of Z and W chromosomes of female **D** C-band of ZZ chromosomes of male **E** Ag-NOR bearing chromosomes of male individuals **F** CMA_3_ treatment of ZZ chromosomes of male **G** FISH 18S rDNA. Bar = 10 µm.

After colloidal silver nitrate treatment, was evidenced only one pair carrier the NOR. This structure is restricted to the terminal region of an acrocentric pair, confirmed by FISH technique (Figure 3 B, E, G). The heterochromatic segments of sex chromosomes, as well as NOR presents bright signals through Chromomycin A_3_ treatment, indicating a GC rich regions (Figure 3 C, F).

## Discussion

In contrast to the situation observed in many families of the order Characiformes, the presence of non conserved karyotype is a common characteristic of Gasteropelecidae species. The diploid numbers range from 2n=48 to 2n=54 chromosomes (Hinegardner and Rosen 1972, Scheel 1973, Terêncio et al. 2008), being mostly, subtelocentric and acrocentric chromosomes (Carvalho et al. 2002).

The *Thoracocharax stellatus* populations analyzed in the present study presented 2n=54 chromosomes in both sexes, similar to the observed for individuals from Araguaia- Tocantins basin (Venere et al. 2007). However, differences related to karyotypic formulae among these populations were observed, as diploid number, frequency of subtelocentric and acrocentric pairs and the presence of multiple NOR system (see Table 1). According to Centofante et al. (2003), cytogenetic studies in fishes have show that populations isolated by geographic barriers are more favourable to the establishment of chromosome alterations. Thus, mechanisms of distance isolation together with historic events have been the principal factors influencing the changes among populations from Amazon and Araguaia-Tocantins basin (Venere et al. 2007), which can also explain the differentiation process observed among the populations from Araguaia and Paraguay rivers.

The NORs have been frequently employed as an important chromosome marker in many fish groups and their changes have been used as an important tool for the identification of chromosome markers among certain species and populations. The *Thoracocharax stellatus* populations studied presented a single NOR system, but a number and size heteromorphysm of these structures was detected in other populations already studied (Table 1). Considering that the karyotypic composition of the Paraguay and Araguaia basins is stable within each population, the variation in number of active sites in these populations can be explained by a possible differential expression of rDNA cistrons as observed in studies of other group of fishes in other basins (Vicari et al. 2006).

According the results with base-specific fluorochromes the heterochromatin observed in *Thoracocharax stellatus* is distributed in blocks containing different proportions of the nucleotides. The first kind, present in few chromosomes of complement, is rich in GC bases. In this group, the heteromorphic chromosomes related to the ZZ/ZW sex system and the NOR chromosomes are included. The W chromosome has a large, CMA_3_+ positive heterochromatic fluorescent segment and in the Z only the pericentromeric regions is evidently heterochromatic. The sex chromosome system in *Thoracocharax stellatus* from the Paraguay basin does not present explicit differences in size between Z and W chromosomes, unlike that observed in populations from other hydrographic basins.

The second kind of heterochromatin, evidenced through C-band technique is characterized by markers located in pericentromeric and telomeric regions of several chromosomes of the complement, without fluorescent signals after the colouring with Chromomycin A_3_. This distribution pattern of heterochromatic blocks was already observed in several studies of chromosome characterization of many groups of fishes, as *Leporinus* (Margarido and Galetti 2000), *Astyanax* (Vicari et al. 2008), *Imparfinis* (Margarido and Moreira-Filho 2008) and *Hypostomus* (Rubert et al. 2008).

The female heterogamety is the most frequently observed sex determination system observed in fishes (Centofante et al. 2002). Depending on the group, the differentiation between Z and W chromosomes can be made by several ways: In some species there is an accumulation of heterochromatic segments followed by an increase of W chromosome in relation to Z chromosome (Centofante et al. 2001), accumulation of heterochromatin followed by loss of DNA sequences (Bertollo and Cavallaro 1992, Carvalho et al. 2002). In other species, substitutions of heterochromatic segments (Centofante et al. 2002) and rearrangements followed or not by loss of heterochromatic material (Artoni et al. 1998, Almeida-Toledo et al. 2002, Rosa et al. 2006). The occurrence of a W chromosome totally heterochromatic in the *Thoracochara*x species studied until now suggests that its origin is related to the accumulation of heterochromatin in these chromosomes.

Phylogenetic relationships based on morphological characters among gasteropelecids and other families have been discussed since Eigenmann (1910) using morphological characters suggested that they are a sister group of Triportheinae and Cynodontidae. The morphological evidence of a possible relationship between Gasteropelecidae and Triportheinae could be enhanced by the description of the similar sex chromosomes in both groups (Artoni and Bertollo 2002, Carvalho et al. 2002, Nirchio et al. 2007, Venere et al. 2007, this study). Some authors suggest that this system is a characteristic established before the speciation of the group *Triportheus* (Artoni et al. 2001, Artoni and Bertollo 2002), so it is acceptable to admit that a similar event may have occurred in the family Gasteropelecidae.

The increase of new cytogenetic studies have shown that other species of Gasteropelecidae such as *Carnegiella marthae*, have 50 chromosomes, NOR polymorphisms and a ZZ / ZW sex differentiation system (Terêncio et al. 2008). In this group, however, the Z and W chromosomes have different size. This variation illustrates the importance of chromosome studies of other species of the family, not cytogenetically characterized yet. The molecular data available to date also reinforce the proximity of *Carnegiella* and *Thoracocharax* genus, according to Abe (2007) based in mtDNA sequence, these two genera together comprises a sister clade of the independent clade composed only of *Gasteropelecus* species.

Isolated events of chromosome rearrangements, without phylogenetic implications, as fission may be responsible to reduction of subtelocentric and increase of larges acrocentric chromosomes, resulting in the chromosomal particularities observed in *Thoracocharax stellatus* from Paraguay Rivers basin in relation to other population already studied. The results also show the cytogenetic diversity of the group, is useful to understand the mechanisms of karyotype evolution of the family as well as understanding the processes of differentiation and evolution of sex chromosomes in the group.
